# Effects of Glycerol and Phenolics on *Myceliophthora heterothallica* Endoxylanase Expressed in *K. phaffii*

**DOI:** 10.3390/biotech14030062

**Published:** 2025-08-18

**Authors:** Jéssica de Araujo Zanoni, Izabela Karolina Costa Zilli, Guilherme de Paula Pretto, Flavio Augusto Vicente Seixas, Marcela Marques de Freitas Lima, Eliana Gertrudes de Macedo Lemos, Eleni Gomes, Gabriel Zazeri, Gustavo Orlando Bonilla-Rodriguez

**Affiliations:** 1 Graduate Program in Microbiology, Institute of Biosciences, Humanities and Exact Sciences, São Paulo State University, IBILCE-UNESP, São José do Rio Preto 15054-000, SP, Brazil; j.zanoni@unesp.br (J.d.A.Z.); izabela.kc.zilli@unesp.br (I.K.C.Z.); g.pretto@unesp.br (G.d.P.P.); 2 Department of Technology, State University of Maringá (UEM), Umuarama 87020-900, PR, Brazil; favseixas@uem.br; 3 Department of Chemistry and Environmental Sciences, Institute of Biosciences, Humanities and Exact Sciences, São Paulo State University (IBILCE-UNESP), São José do Rio Preto 15054-000, SP, Brazil; marcela-marques.lima@unesp.br (M.M.d.F.L.); eleni.gomes@unesp.br (E.G.); 4 Department of Technology, School of Agricultural and Veterinarian Sciences, São Paulo State University (FCAV-UNESP). Jaboticabal 14884-900, SP, Brazil; eliana.lemos@unesp.br; 5 Department of Physics, Federal University of Roraima (UFRR), Boa Vista 69310-000, RR, Brazil

**Keywords:** rMhXyn, glycerol stabilization, enzyme kinetics, thermostability, biophysical chemistry, *Komagataella phaffii* expression

## Abstract

Industrial applications of xylanases in high-temperature settings are limited by enzyme instability. This study evaluated glycerol and phenolic compounds as modulators of the catalytic and structural properties of a recombinant *Myceliophthora heterothallica* endoxylanase (rMhXyn) expressed in *Komagataella phaffii*. Glycerol (20% *v*/*v*) significantly improved thermostability (5-fold increase in half-life at 55 °C), decreased the activation energy for catalysis, and enhanced structural rigidity as evidenced by molecular dynamics simulations (reduced RMSD and Rg). In contrast, phenolic acids provided only short-term stabilization at moderate temperatures and did not confer structural benefits. Enzyme kinetics revealed that glycerol enhanced catalytic turnover (↑*V*_max_), while phenolic compounds modified both *K*′ and cooperativity (Hill coefficient). Thermodynamic analysis supported glycerol’s stabilizing effect, with increased ∆*H*_(D)_ and a positive shift in ∆*S*_(D)_. These results suggest glycerol as a superior stabilizer for rMhXyn in high-temperature bioprocesses such as lignocellulosic biomass hydrolysis. These findings highlight the potential of targeted additives to improve enzyme performance for biotechnological applications.

## 1. Introduction

The degradation of xylan, the principal polysaccharide component of plant cell wall hemicellulose, requires the coordinated action of a suite of enzymes collectively referred to as the “xylanolytic complex.” Central to this complex are endo-1,4-β-xylanases (EC 3.2.1.8), which catalyze the hydrolysis of β-1,4 glycosidic linkages within the xylan backbone, releasing xylooligosaccharides and monomeric sugars with broad biotechnological applications [1]. These enzymes are produced by a diverse range of organisms, including filamentous fungi, macrofungi, bacteria, seaweeds, and certain germinating plant seeds. Structurally, they adopt a canonical β-barrel fold and utilize conserved glutamate residues as catalytic acid/base groups, underpinning their functionality and industrial relevance [2,3]. The biotechnological potential of endoxylanases spans multiple industrial processes. They enable the production of dietary sweeteners with low caloric value, as well as xylooligosaccharides (XOS) with prebiotic properties that promote the growth of *Bifidobacterium* spp. and *Lactobacillus* spp. in the gastrointestinal tract, thereby reducing colon inflammation and the risk of colon cancer. These compounds are also used in the pharmaceutical industry [4,5,6,7]. In paper manufacturing, their low molecular mass allows penetration of cellulose fibers, removing the hemicellulose fraction from pulps, which results in enhanced pulp bleaching and reduced chlorine use [8]. In animal feed supplementation, they improve nutrient availability for poultry and enhance forage digestibility for ruminants [9,10]. A promising application lies in second-generation biofuels production, such as ethanol, through the fermentation of monosaccharides released by the hydrolytic action of these enzymes on agro-industrial by-products [11,12].

Despite their industrial versatility, xylanase performance is often challenged by environmental factors in industrial settings. Phenolic compounds, abundant in lignocellulosic biomass, can modulate enzyme activity through covalent and non-covalent interactions that are strongly influenced by the chemical structure of each phenolic compound [13].

Glycerol, a commonly used stabilizer in enzyme formulations, is well recognized for its ability to enhance protein thermostability; however, its effects on the kinetics and structural dynamics of xylanases remain insufficiently characterized [14,15]. Additionally, the observation of cooperative behavior in monomeric enzymes, such as the *Myceliophthora heterothallica* endoxylanase, offers a unique opportunity to uncover novel regulatory mechanisms [16].

This study aims to address these knowledge gaps by investigating the influence of glycerol and selected phenolic compounds (4-hydroxybenzoic, ferulic, vanillic, and syringic acids) on the kinetics, thermodynamics, and structural properties of a recombinant *Myceliophthora heterothallica* endoxylanase expressed in *Komagataella phaffii*. Employing a multidisciplinary approach that integrates in silico analyses—including bioinformatics sequence analysis, structural modeling, molecular docking, and molecular dynamics simulations—with experimental assays, we seek to elucidate the modulatory effects of these compounds on enzyme performance and stability. The findings are anticipated to provide valuable insights for the optimization of xylanase applications in industrial processes.

## 2. Materials and Methods

### 2.1. In Silico Analysis

#### 2.1.1. Sequence Analysis

De Amo and colleagues [17] reported the amino acid sequence, which is publicly available in GenBank under accession number MK204363. This sequence was subjected to verification for identity and the identification of non-functional regions, including transit and signal peptides, using the UniProt server. Subsequently, the region corresponding to the xylanase domain (UniProt ID: G2QNI1) was analyzed with the NetNGlyc-1.0 server to predict potential N-glycosylation sites. To identify suitable structural templates, particularly those of glycosylated xylanases, the sequence was further subjected to BLASTP (NCBI, version 2.17.0) analysis against the Protein Data Bank (PDB) database.

#### 2.1.2. Structural Modeling

The unique features of the sequence were analyzed, and the three-dimensional structure of the xylanase was modeled using AlphaFold 2 (Google DeepMind, London, UK) and RoseTTAFold (Baker Lab, University of Washington, Seattle, WA, USA). The sequence numbering was adjusted to correspond with the previously obtained UniProt entry, beginning at S1 and terminating at A200. In the resulting three-dimensional model, the first residue is V5.

N-glycosylation at N69, predicted using NetNGlyc-1.0, was modeled with Glycan.com based on an oligomannose structure classified as a high-mannose-type glycan. The potential interactions between the glycan atoms and the substrate were assessed within a 4 Å distance using PyMOL (Schrödinger lnc., New York, NY, USA).

#### 2.1.3. Molecular Docking of Phenolic Compounds

The *Myceliophthora heterothallica* endoxylanase structure, modeled with glycosylated oligosaccharides, was employed in blind docking simulations using Molegro Virtual Docker v6.01. Prior to docking, all water molecules and salt ions were removed from the structure. Cavities within the polypeptide chain were identified utilizing the expanded van der Waals method, with cavity volumes ranging from 5 to 100,000 Å^3^, a probe radius of 1.2 Å, and a grid resolution of 0.80 Å. The docking search was centered at coordinates (4.50, –9.04, –21.59) with a search radius of 35 Å. The MolDock Simplex Evolution algorithm was selected as the search function, and the MolDock Score [GRID] was used for ranking, both with default parameters. The compounds’ three-dimensional structures were obtained from the PubChem database in *.sdf format and were used as a library in five simulations to obtain ten poses per simulation of each compound mentioned in Figure 5A of the article from Massarente et al. [18], in addition to tannic acid. The scores of the five best poses in each cavity, sorted by Rerank Score, were used to calculate the mean score.

#### 2.1.4. Investigating the Rapid Onset of Glycerol’s Stabilizing Action via Molecular Dynamics

Molecular dynamics (MD) simulations were performed in order to assess the immediate structural impact of glycerol on the enzyme’s structure. Although the 1 ns simulation time restricts extensive sampling of the conformational landscape, it was considered sufficient for the primary objective of detecting early-onset differences in structural integrity and compactness. Potential structural perturbations induced by glycerol were assessed by comparing the root mean square deviation (RMSD) of atomic positions and radius of gyration (Rg) values obtained from molecular dynamics simulations of the enzyme both in isolation and in the presence of glycerol. The structure of *Neocallimastix patriciarum* xylanase, resolved by X-ray diffraction at 1.80 Å resolution and available in the Protein Data Bank (PDB ID: 2VGD) complexed with two repetitive substrate units in the active site [19], was employed as a model for the closed (active) conformation. Molecular dynamics simulations of the 2VGD complex, solvated with explicit SPC water molecules (10,265 molecules), were performed for 1 nanosecond (ns) using the AMBER 99 force field [20] under periodic boundary conditions. The cubic simulation cell volume was 360.546 nm^3^. Detailed information regarding the simulation setup and parameters is provided in Part I [21]. Trajectory analyses and molecular structure evaluations were conducted using the Visual Molecular Dynamics software VMD, version 1.9.3 (University of Illinois at Urbana-Champaign, Urbana, IL, USA) [22]. Various geometric parameters indicated that the protein system reached equilibrium within less than 1 ns. Simulations were initiated from the 2VGD crystallographic structure and subjected to steepest descent energy minimization until no significant energy change could be detected (<0.01 kJ.mol). This was followed by a 5-picosecond (ps) initialization run, with initial velocities taken from a Maxwellian distribution and using a temperature coupling constant of 0.01 ps and a pressure coupling constant of 0.05 ps [23]. The non-bonded pair list was updated every 10 femtoseconds (fs), and non-bonded interactions were truncated at 8 and 10 Å, respectively. The SHAKE algorithm [24] was applied to constrain bond lengths to their equilibrium positions, and the equations of motion were solved using the Verlet algorithm [25]. Simulations were performed with a 2-fs time step, and coordinates were saved every 0.05 ps.

### 2.2. Enzyme Characterization

The cloning, expression, and purification of the rMhXyn were performed following the methods outlined by de Amo et al. [16], adding two procedures. Purification included ethanol precipitation and size-exclusion chromatography in Sephadex G-50 and Superdex 75, resulting in a 13.7-fold purification and specific activity of 92.4 U mg^−1^. The enzyme retained the His tag and the TEV protease cleavage site.

#### 2.2.1. Reagents and Materials

The following materials were used in the experimental assays. Beechwood xylan (substrate for enzymatic activity), 3,5-dinitrosalicylic acid (DNS), and ADA buffer components were purchased from Sigma-Aldrich (St. Louis, MO, USA). The phenolic acids (4-hydroxybenzoic, ferulic, vanillic, and syringic acids) and glycerol (≥99.5%) were acquired from Merck (Darmstadt, Germany). All solutions were prepared using ultrapure water (ELGA LabWater, Lane End, UK), and pH adjustments were performed with NaOH or HCl as needed.

#### 2.2.2. Enzyme Assays

To measure the endoxylanolytic activity, the sample was mixed with 1% (w/v) beechwood xylan, which was diluted in 0.1 mol·L^−^^1^ ADA buffer pH 6.0, and exposed to 65 °C for 5 min. The resulting reducing sugars reacted with 3,5-dinitrosalicylic acid (DNS), and the activity was quantified by measuring the change in product concentration over time (d[P]/dt) [26] using spectrophotometry. The incubation time was defined based on experiments to ensure linear product release over time. All experiments were conducted in triplicate. The initial reaction rate (Vo) in µmol·min^−1^·mg^−1^ was calculated at different substrate concentrations (ranging from 1 to 10 mg·mL^−1^).

To describe the kinetic behavior, nonlinear regression analysis was performed using the QtiPlot program version 0.9.9.11 (©Ion Vasilief 2004–2017) for Linux, applying the Hill equation. This model was used after we attempted to fit the classical Michaelis–Menten model, which proved inadequate due to the sigmoidal behavior observed in the saturation curve, suggestive of cooperative substrate binding. The values were determined through nonlinear parameter fitting using the Hill equation derived from the Michaelis–Menten equation, where the semisaturation constant *K′* and [S] are raised to the exponent “*h*”, cooperativity [27]:

V_o_ = (*V*_max_ × [S]^h^)/(*K′*^h^ + [S]^h^) (1)

The turnover number *K*_cat_ was calculated from *V*_max_ and the enzyme concentration, and, subsequently, *k*, the catalytic efficiency (*k*_cat_/*K′*).

#### 2.2.3. Pre-Incubation of the Enzyme with Substances to Be Evaluated

To evaluate the effects of phenolic acids, namely 4-hydroxybenzoic acid, ferulic acid, vanillic acid, and syringic acid, on the kinetics of rMhXyn, the enzyme was pre-incubated with these compounds at a final concentration of 20 mmol·L^−1^. Additionally, to investigate the effects of glycerol on kinetics and thermodynamics, the enzyme was exposed to a final concentration of 20% (*v*/*v*) glycerol. Control treatments were also included, using ultrapure water in the same proportion as the compounds and glycerol. After mixing, the samples were allowed to rest at room temperature for 30 min before determining their kinetic and thermodynamic parameters, which are described below.

#### 2.2.4. Thermodynamic Parameters

The thermodynamic analysis of rMhXyn’s thermal denaturation was conducted using Beechwood xylan at the optimal pH. The thermodynamic parameters, which included the activation energy (E_a_), temperature coefficient (Q_10_), half-life (T_1/2_), and other enzyme-related parameters related to thermal denaturation such as activation energy E_a(D)_, Δ*H*_(D)_, Δ*G*_(D)_, and Δ*S*_(D)_, were determined using a method proposed in the literature [28,29] and previously employed in studies on other enzymes [30,31]. The irreversible denatured “I” state was evaluated using the N ↔ D → I model, where “N” represents the native conformation, and “D” represents the reversible denatured conformation.

To analyze the effect of phenolic compounds on the thermal stability of the enzyme, xylanase was incubated with the studied compounds at different temperatures (50, 60, 70, and 75 °C) for 30, 60, 90, and 120 min, for its residual activity to be determined [28,29,32].

## 3. Results and Discussions

### 3.1. Sequence and Structural Analysis in Silico

**Figure 1:**
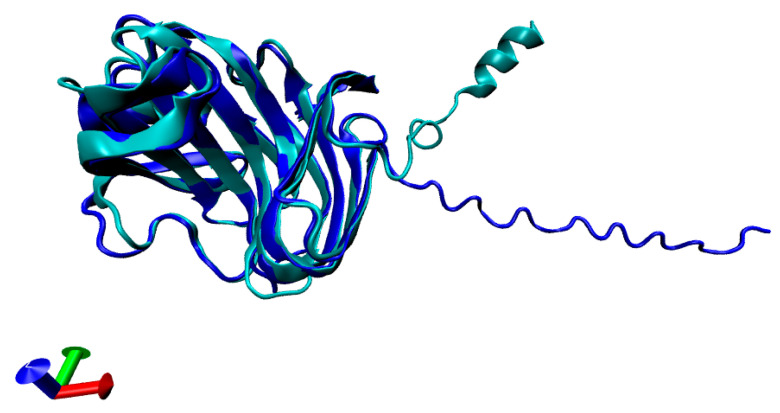
Structural modeling of *Myceliophthora heterothallica* endoxylanase models obtained with Alphafold 2 (in blue) and RoseTTaFold (in cyan), highlighting the β-barrel fold.

Using BLAST against the UniProtKB database, the G2QNI1 entry was identified with 100% identity, corresponding to the endo-1,4-beta-xylanase from Myceliophthora heterothallica (strain ATCC 42464/BCRC 31852/DSM 1799) (also known as Sporotrichum thermophile). The full-length sequence includes a start codon (methionine), a signal peptide, and the xylanase domain. Structural models generated by AlphaFold 2 (blue) and RoseTTAFold (cyan) demonstrated excellent structural overlap (Figure 1), both exhibiting the characteristic β-barrel fold typical of GH11 xylanases [33]. The signal peptide spans residues 2 to 18, while the xylanase domain spans residues 19 to 218. 

**Figure 2:**
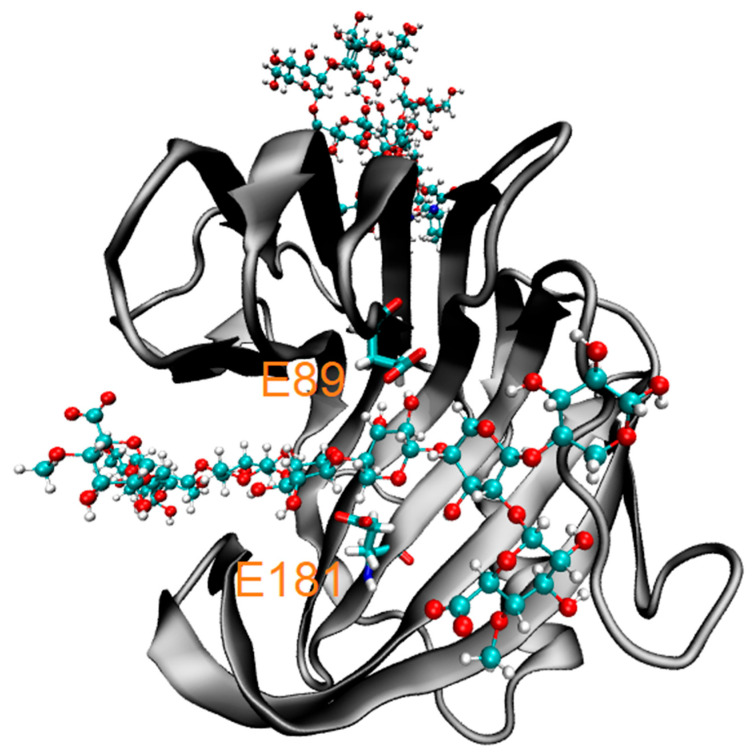
Representation of the glycosylated structural model of the endoxylanase from *Myceliophthora heterothallica*, observing the catalytic residues E89 and E181 close to the substrate in the active site, and the N69 glycosylation located opposite the active site of the enzyme.

N-glycosylation at N69 was predicted using NetNGlyc-1.0 and modeled with Glycan.com, employing an oligomannose structure classified as a high-mannose-type glycan. Structural analysis using PyMOL revealed no interactions between the glycan atoms and the substrate within a 4 Å radius (Figure 2). Only residues in proximity to the enzyme’s active site exhibited potential interactions with the substrate. The residues identified were Q7, T10, S19, W21, N47, V49, N74, Y78, Y80, W82, E89, Y99, T123, R125, P129, S130, I131, G133, Y175, E181, and Y183. Among these, E89 and E181 were determined to function as the catalytic residues at the enzyme’s cleavage site (Figure 2).

### 3.2. Enzyme Kinetics

#### 3.2.1. Effect of Phenolics on Enzymatic Kinetics

**Figure 3:**
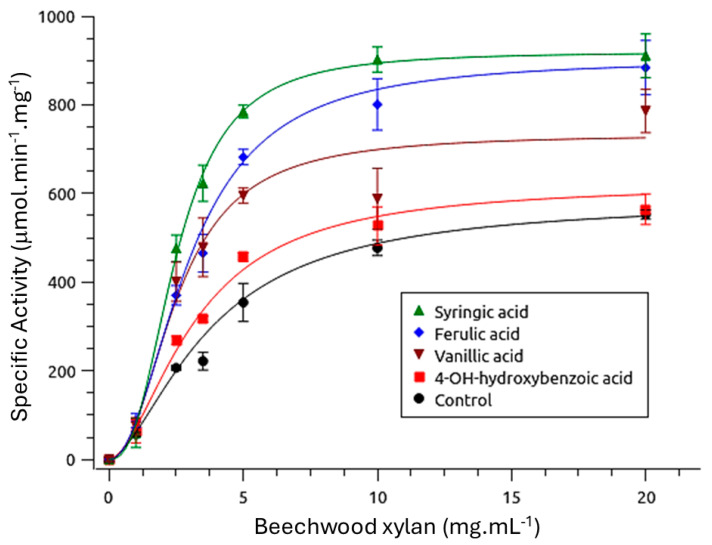
Kinetic parameters of *Myceliophthora heterothallica* endoxylanase expressed in *Komagataella phaffii*, evaluated using beechwood xylan as a substrate at concentrations ranging from 1 to 20 mg·mL^−1^, both with and without phenolic compounds. Assays were conducted at the enzyme’s optimum pH with an initial reaction time of 1 min. The lines represent the fitted curves based on the Hill equation, and error bars indicate the standard deviation (S.D.) from triplicate experiments.

Although the enzyme is monomeric [16], the sigmoidal kinetic curves presented in Figure 3 indicate cooperative behavior, even in the absence of phenolic compounds. The presence of vanillic, syringic, and ferulic acids significantly reduced the K′ values (*p* < 0.05), whereas 4-hydroxybenzoic acid did not produce a statistically significant effect (*p* > 0.05) (Table 1). With the exception of 4-hydroxybenzoic acid (*p* > 0.05), all phenolic compounds increased the *V*_max_. Additionally, cooperativity values (Hill coefficient, h) increased significantly (*p* < 0.05) in the presence of syringic and ferulic acids.

The rMhXyn displays cooperative kinetic behavior, as evidenced by sigmoidal activity curves and Hill coefficients (*h*) greater than 1, despite its monomeric structure. This homotropic allosteric behavior—where substrate binding enhances the affinity for subsequent substrate molecules—resembles that observed in glucokinase (h ≈ 1.7), and is attributed to slow conformational transitions between high- and low-affinity states, as described by the mnemonic model [34]. Such behavior implies that the enzyme transitions between distinct conformational states, allowing for a finely regulated catalytic response to varying substrate concentrations. 

Phenolic compounds enhance the enzyme’s catalytic performance, primarily by increasing *V*_max_ and, in some cases, reducing *K*′ (Table 1). These effects, consistent with prior studies [35,36], arise from non-covalent interactions between phenolic hydroxyl or methoxyl groups and aromatic amino acid residues in the enzyme. The chemical structure of phenolics, particularly the number and position of these groups, determines whether they have enzyme affinity and conformational stability [13].

#### 3.2.2. Molecular Docking of Phenolics

**Figure 4:**
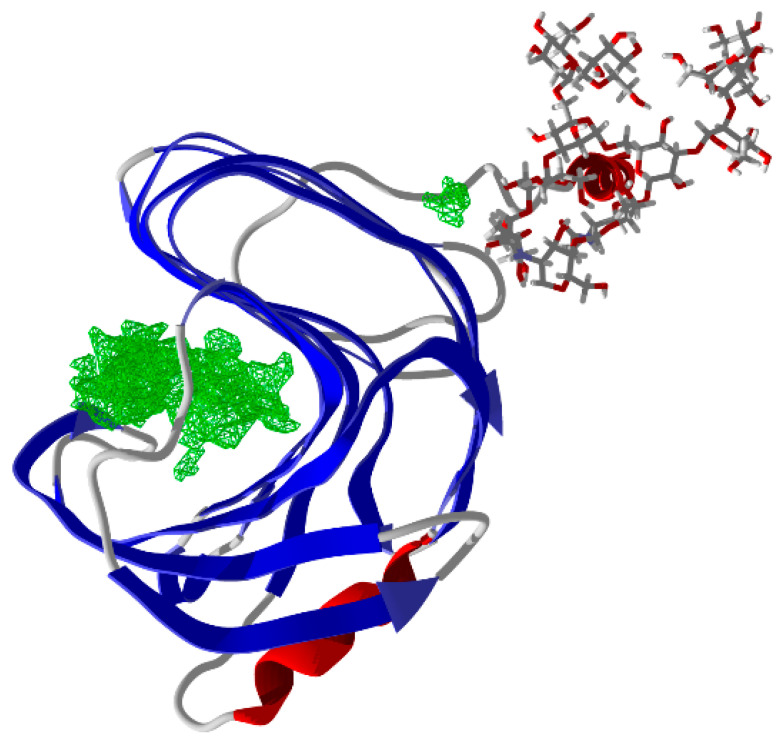
Identification of the active site cavity (195.0 Å^3^) in the *Myceliophthora heterothallica* endoxylanase structure with N-terminal His-tag, visualized using Molegro Virtual Docker.

The search for cavities in the three-dimensional structure revealed only one large cavity (195.0 Å^3^) corresponding to the enzyme’s active site (Figure 4). No additional cavities were identified as possible sites for allosteric ligands. The binding modes of four ligands (4-hydroxybenzoic, ferulic, vanillic, and syringic acids) were investigated through molecular docking with AutoDock Vina. Five simulations were run for each ligand, and the top ten poses from each simulation were collected, leading to a total of 50 poses per ligand.

Preliminary results (not shown) indicated weak interactions between the phenolic compounds and the enzyme, suggesting that these molecules are unlikely to remain stably bound within the identified cavity. Molecular docking simulations supported this observation, revealing weak and non-specific binding, thereby excluding allosteric modulation as the primary mechanism of enzymatic enhancement. Instead, we propose that phenolic compounds adsorb onto xylan fibers, thereby increasing substrate accessibility. This adsorption process is likely rapid and pH-dependent, as phenolic compounds dissociate in aqueous media, releasing protons and phenolate anions (PhO^−^) [37]. At the enzyme’s optimal pH—above the pKa values of the phenolic acids evaluated—phenolate formation enhances electrostatic attraction to xylan fibers, potentially facilitating enzyme–substrate (ES) complex formation, as evidenced by the observed decreases in *K*′ and increases in *V*_max_ (Table 1).

Recent studies have demonstrated that the adsorption of ferulic acid onto xylan decreases with increasing pH, further supporting this proposed mechanism [38]. These findings suggest that phenolic compounds may improve xylanase performance during lignocellulosic biomass processing, which is of particular relevance for second-generation biofuel production. Nonetheless, the adsorption-based mechanism remains hypothetical and warrants further validation through experimental techniques such as spectrofluorometry, Fourier-transform infrared (FT-IR) spectroscopy, circular dichroism spectroscopy, and ultraviolet absorption analysis, to elucidate the nature of phenolic compound interactions with the ES complex [39]. Molecular docking provides a preliminary framework to support this hypothesis and may guide future efforts aimed at optimizing xylanase performance in biotechnological applications.

#### 3.2.3. Effect of Glycerol on Enzyme Kinetics

The effect of glycerol (20% *v*/*v*) on the rMhXyn’s kinetics was evaluated using enzymatic assays with beechwood xylan as the substrate. Glycerol significantly increased the maximum reaction rate ((*V*_max_): 699.9 ± 19.1 µmol·min^−1^·mg^−1^ vs. 526.6 ± 1.20 µmol·min^−1^·mg^−1^ in control, *p* < 0.05) and reduced the Hill coefficient ((*h*): 1.85 ± 0.18 vs. 2.79 ± 0.2, *p* < 0.05), with no significant change in the semisaturation constant ((*K′*): 2.36 ± 0.11 mg·mL^−1^ vs. 2.41 ± 0.07 mg·mL^−1^, *p* = 0.53), turnover number ((*K_cat_*): 32.48 ± 0.89 vs. 24.44 ± 0.05 s*^−^*^1^, *p* > 0.05) and catalytic efficiency ((*K*_cat_/*K*′)): 13.77 ± 0.27 vs. 10.15 ± 0.26, *p* > 0.05) (Table 2). The unchanged *K′* indicated that glycerol did not enhance substrate affinity, while the elevated *V*_max_ suggests improved catalytic turnover, potentially due to stabilization of the active-site conformation. The reduced h implies a slight decrease in cooperative behavior, contrasting with phenolic compounds, which enhance cooperativity (Section 3.2.1).

Glycerol was found to reduce the Hill coefficient, indicating the stabilization of a specific catalytic conformation and a consequent narrowing of the enzyme’s conformational ensemble. This effect is supported by an increase in the maximum reaction velocity (*V*_max_) without significant changes in substrate affinity (*K*′), suggesting enhanced catalytic efficiency. When compared to a recombinant endo-1,4-β-xylanase from *Penicillium occitanis* expressed in *Komagataella phaffii*, which exhibited a *K*_m_ of 8.33 ± 0.7 mg·mL^−1^ and a *V*_max_ of 58.82 ± 0.9 µmol·min^−1^·mg^−1^ using oat spelt xylan at pH 3.0 and 50 °C [40], rMhXyn demonstrates superior substrate affinity and catalytic activity, highlighting its potential for industrial applications. Notably, similar cooperative kinetics have been observed in other GH11 xylanases, including those from *Bacillus subtilis* [41], further supporting the distinct nature of this monomeric allosteric behavior.

### 3.3. Thermodynamics

#### 3.3.1. Effect of Phenolic Compounds on Thermal Stability

**Figure 5:**
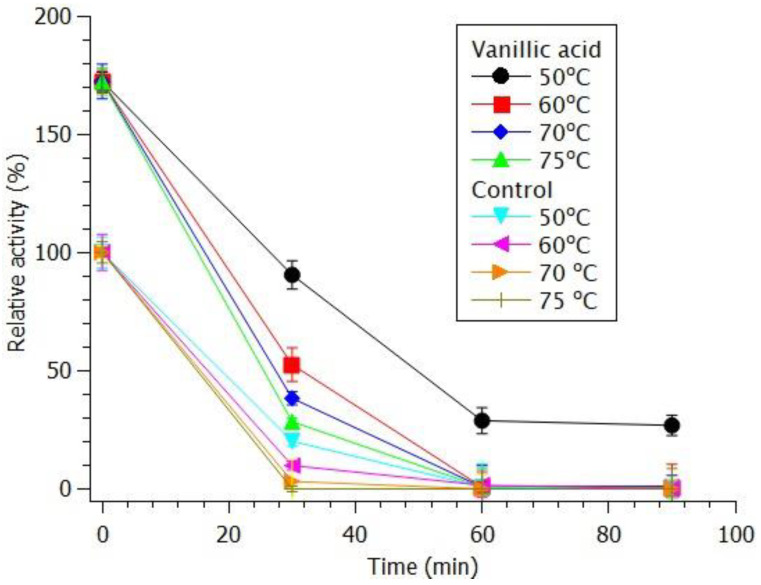
Residual activity of *Myceliophthora heterothallica* endoxylanase expressed in *Komagataella phaffii* pre-incubated with 20 mM vanillic acid at 50–75 °C over 0–90 min.

The phenolic compounds previously employed in the kinetic assays were evaluated for their effects on enzyme stability under thermal stress at 50, 60, 70, and 75 °C, with incubation periods of 0, 30, 60, and 90 min. With the exception of 4-hydroxybenzoic acid, all tested phenolic compounds maintained enzymatic activity above that of the control up to the final time point at 50 °C. Notably, at the 30 min mark, enzyme activity was consistently higher in the presence of the phenolic compounds; however, beyond 60 min, no significant differences were observed, and enzymatic activity was no longer detectable. Figure 5 illustrates the representative thermal stability profile observed for vanillic acid.

#### 3.3.2. Effect of Glycerol on Thermal Stability

The thermal stability of the rMhXyn was assessed under optimal pH conditions (pH 6.0) at temperatures ranging from 55 to 70 °C, with and without 20% glycerol. The estimated value was 66.0 ± 0.5 °C in both conditions (Figure S1 in the Supplementary Materials). The addition of 20% glycerol lowered the enzyme’s activation energy (E_a_) from 50.9 to 36.2 kJ·mol^−1^ (Equation (S2) in the Supplementary Materials). This 1.4-fold reduction in the energy barrier indicates enhanced catalytic efficiency, a finding consistent with the increased maximum velocity (*V*_max_) observed in kinetic assays (Section 3.2.3).

The Q_10_ (Equation (S3) in the Supplementary Materials), reflecting the reaction rate increase per 10 °C, was reduced with glycerol (Table 3), suggesting reduced sensitivity of the protein structure to thermal fluctuations. Glycerol extended the enzyme’s half-life (Equation (S4) in the Supplementary Materials) approximately 2-fold at 60–75 °C and 5-fold at 55 °C (Table 3).

Thermodynamic parameters further corroborate the stabilizing effect of glycerol on the enzyme’s structure, as summarized in Table 4. The activation energy for denaturation (E_a(*d*)_) (Equation (S5) in the Supplementary Materials) increased substantially in the presence of glycerol, rising from 59.86 to 119.47 kJ·mol^−1^, effectively doubling the energy required for denaturation. Similarly, the enthalpy (Equation (S6) in the Supplementary Materials) change for denaturation (∆*H_D_*) at 55 °C increased from 57.13 kJ·mol^−1^ in the control condition to 116.75 kJ·mol^−1^ in the presence of glycerol. At 70 °C, a slight decrease was observed, with ∆*H_D_* values of 57.01 and 116.62 kJ·mol^−1^ for the control and glycerol-treated samples, respectively, reflecting a minimal reduction of approximately 0.1 kJ·mol^−1^ per 15 °C.

The ∆*G*_D_ (Equation (S7) in the Supplementary Materials) exhibited a modest increase in the presence of glycerol, rising by approximately 5% from 93.78 to 98.21 kJ·mol^−1^ at 55 °C, and by 2% from 95.52 to 97.73 kJ·mol^−1^ at 70 °C. In contrast, ∆*S*_D_ (Equation (S8) in the Supplementary Materials) demonstrated a pronounced shift, transitioning from negative values under control conditions (−110.72 J·mol^−1^·K^−1^ at 55 °C and −112.26 J·mol^−1^·K^−1^ at 70 °C) to positive values in the presence of glycerol (56.50 J·mol^−1^·K^−1^ and 55.09 J·mol^−1^·K^−1^, respectively). These changes further support the role of glycerol in stabilizing protein folding and maintaining structural integrity.

Glycerol markedly enhances both the catalytic turnover and structural stability of rMhXyn. This enhancement may result from a narrowing of the enzyme’s conformational ensemble, thereby reducing the Hill coefficient (*h*) by limiting transitions between distinct conformational states (e.g., E_1_ and E_2_), as described in models of monomeric cooperativity [42]. The increase in *V*_max_ without a corresponding change in *K*′ indicates that glycerol improves catalytic efficiency without affecting substrate affinity. This enhancement translates into improved xylanase performance under conditions relevant to lignocellulosic biomass processing, a critical step in second-generation biofuel production [12].

The observed effects are likely attributable to glycerol’s interaction with the enzyme’s hydration shell, promoting the retention and structuring of water molecules around the protein. This interaction contributes to the stabilization of the enzyme’s native conformation (Section 3.1) and improved catalytic activity [14,15,43], as further supported by molecular dynamics (MD) simulations (Section 3.3.3).

Thermodynamic analyses further substantiated glycerol’s stabilizing role. The activation energy for denaturation (E_a(D)_) and enthalpy of denaturation (Δ*H*_D_) were significantly higher in the glycerol condition, indicating that more energy is required to unfold the enzyme. The higher Δ*H*_D_ and Δ*G*_D_ with glycerol reflect increased energy barriers to denaturation, preserving hydrogen bonds and van der Waals interactions [44]. The positive ΔS_D_ shift suggests greater disorder in the unfolded state, likely due to reduced water structuring around exposed hydrophobic residues [45,46,47], contrasting with the control’s negative entropy, which may result from solvent reorganization in clathrates. These changes align with glycerol’s known osmolyte properties. The pronounced shift in Δ*S*_D_ suggests an entropic stabilization mechanism, possibly due to disruption of organized water structures and reduced configurational entropy in the folded state. This aligns with glycerol’s osmolyte effect, enhancing stability without altering substrate affinity. Unlike phenolic compounds, which enhance short-term stability at 50 °C (Section 3.3.1), glycerol provides sustained thermostability, critical for high-temperature bioprocessing such as lignocellulosic biomass hydrolysis for second-generation biofuel production [48].

#### 3.3.3. Molecular Dynamics and Glycerol Effect

**Figure 6:**
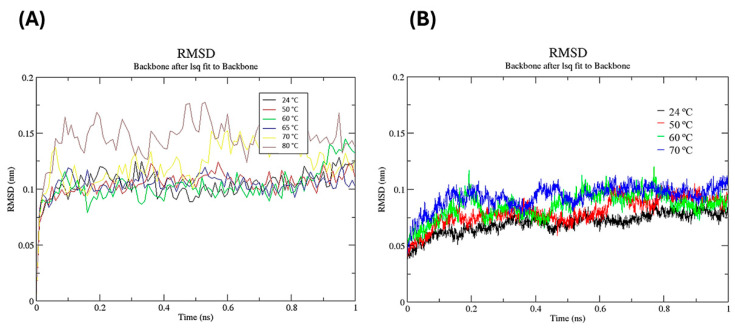
Root mean square deviation (RMSD) of Myceliophthora heterothallica endoxylanase during 1 ns molecular dynamics simulations at 24–80 °C. (**A**) Free enzyme. (**B**) With 20% (*v*/*v*) glycerol.

MD simulations of the *Myceliophthora heterothallica* endoxylanase were performed using the AMBER99 force field. To assess the immediate structural impact of glycerol across various temperatures, we conducted 1 ns simulations at 24, 50, 60, 65, 70, and 80 °C. The simulations were compared in the presence and absence of a 20% (*v*/*v*) glycerol concentration. The results corroborate the thermostability profile described in Section 3.3.2, demonstrating glycerol’s stabilizing effect. This was evidenced by reduced structural fluctuations (lower RMSD) and enhanced compactness (lower Rg values) observed in the simulations containing glycerol compared to those without it. For the free enzyme (Figure 6A), the RMSD value increased rapidly during the first 0.1 ns, reaching 0.1 nm at 24, 50, 60, and 65 °C, fluctuating consistently throughout the simulation. Only at 70 and 80 °C did the RMSD values increase further, showing considerable fluctuation after 0.1 ns, with values nearing 0.15 nm. In the presence of glycerol (Figure 6B), the RMSD values reduced at all evaluated temperatures, reaching stability at 0.2 ns and fluctuating around 0.075 nm, while the free enzyme fluctuated around 0.1 nm (Figure 6A).

**Figure 7:**
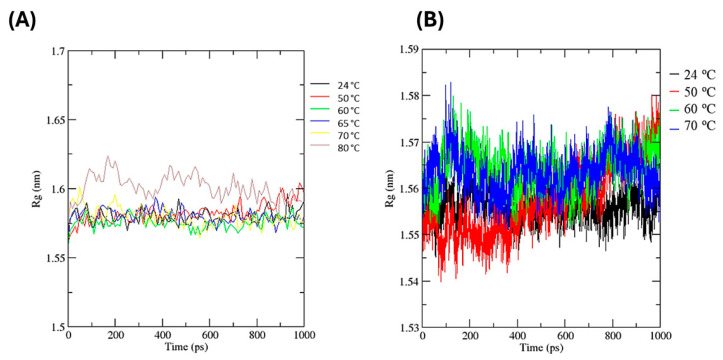
Radius of gyration (Rg) of Myceliophthora heterothallica endoxylanase during 1 ns molecular dynamics simulations at 24–80 °C. (**A**) Free enzyme. (**B**) With 20% (*v*/*v*) glycerol.

The radius of gyration (Rg) values were slightly lower in the presence of glycerol (Figure 7B), averaging 1.56 nm, compared to the free enzyme (Figure 7A), which averaged approximately 1.58 nm. At temperatures of 60, 65, and 70 °C, similar fluctuations in Rg were observed over the simulation period for the free enzyme. Comparable trends were observed for the enzyme in the presence of glycerol at 60 and 70 °C. In both conditions, Rg values at 24 °C remained relatively stable throughout the simulation, whereas at 50 °C, a notable increase occurred during the final 200 ps. Of particular interest is the maximum Rg value recorded at 70 °C, where the free enzyme reached 1.60 nm, compared to a slightly lower maximum of 1.58 nm in the presence of glycerol.

The MD data demonstrated reduced root mean square deviation (RMSD 0.075 nm) and radius of gyration (Rg 1.56 nm) in the presence of glycerol, suggesting a more compact, stable enzyme structure. These findings align with RMSD values reported for other GH11 xylanases from *Bacillus subtilis* (0.15 nm at 25 °C) [43] and the GH10 xylanase from *Thermotoga maritima* (0.1–0.15 nm at 90 °C) [49], indicating enhanced resistance to thermal denaturation. The RMSD variability at higher temperatures may stem from increased atomic fluctuations as the enzyme approaches thermal denaturation. In control simulations, the absence of glycerol likely permits greater conformational flexibility, explaining the elevated RMSD noise. Glycerol, acting as a crowding agent, dampens these fluctuations. These structural observations are consistent with the biochemical data, which showed increased enzymatic stability and activity in the presence of glycerol, highlighting a strong correlation between molecular dynamics and functional behavior.

## 4. Conclusions

The recombinant *Myceliophthora heterothallica* endoxylanase (rMhXyn) demonstrates strong potential as a biotechnological tool for high-temperature industrial processes, with optimal activity at pH 6.0 and 66 °C. While phenolic compounds provided only limited short-term stabilization, glycerol (20% *v*/*v*) significantly enhanced enzyme thermostability—prolonging half-life, decreasing activation energy for catalysis, and increasing both the enthalpy and entropy of denaturation. Molecular dynamics simulations revealed reduced structural fluctuations and a more compact conformation, consistent with the osmoprotective role of glycerol. These findings highlight glycerol as a superior stabilizer, improving rMhXyn’s catalytic efficiency and structural resilience. Overall, the enzyme’s robust thermal profile and responsiveness to biocompatible additives reinforce its applicability in biotechnological processes, particularly in lignocellulosic biomass hydrolysis for second-generation biofuel production and other bio-based industrial platforms.

## Figures and Tables

**Table 1:** Kinetic parameters of *Myceliophthora heterothallica* endoxylanase expressed in *Komagataella phaffii* in the presence of 10 mmol·L*^−^*^1^ of phenolic compounds (beechwood xylan, pH 6.0, 65 °C).

	*K′* (mg·mL^−1^)	*V*_max_ (µmol·min^−1^·mg^−1^)	*h*	*K_cat_* (s^−1^)	*K_cat_*/*K′* (M^−1^·s^−1^)
Syringic acid	2.50 ± 0.07	919.30 ± 16.29	2.57 ± 0.16	42.71 ± 0.83	17.08 ± 0.15
Ferulic acid	2.99 ± 0.19	903.44 ± 43.44	2.07 ± 0.15	41.94 ± 2.02	14.03 ± 0.22
Vanillic acid	2.54 ± 0.30	733.31 ± 52.00	2.20 ± 0.30	34.03 ± 2.40	13.44 ± 0.63
4-hydroxybenzoic acid	3.18 ± 0.62	620.91 ± 100.07	1.76 ± 0.50	28.81 ± 4.64	9.10 ± 0.31
Control	3.72 ± 0.34	583.60 ± 25.56	1.64 ± 0.20	27.08 ± 1.19	7.3 ± 0.34

**Table 2:** The kinetic parameters of *Myceliophthora heterothallica* endoxylanase, expressed in *Komagataella phaffii*, were determined using beechwood xylan as a substrate. The assays were conducted at pH 6.0 and 65 °C in the presence of 10 mmol·L^−1^ of the substrate and 20% (*v*/*v*) glycerol.

	*K′* (mg·mL^−1^)	*V_max_* (µmol·min^−1^·mg^−1^)	*h*	*K*_cat_ (s^−1^)	*K*_cat_/*K′* (M^−1^·s^−1^)
Glycerol	2.36 ± 0.11	699.9 ± 19.1	1.85 ± 0.18	34.48 ± 0.89	13.77 ± 0.27
Control	2.41 ± 0.07	526.6 ± 1.20	2.79 ± 0.20	24.43 ± 0.05	10.15 ± 0.26

**Table 3:** Temperature coefficients (Q_10_) and kinetic parameters of the irreversible denaturation terms of *Myceliophthora heterothallica* endoxylanase expressed in *Komagataella phaffii* estimated based on the first-order Arrhenius plot.

Temperatura		Control			20% Glycerol		
°C	K	Q_10_	*k_d_* (min^−1^)	T _½_ (min)	Q_10_	*k_d_* (min^−1^)	T _½_ (min)
55	313.15	1.77	0.00793	87.39	1.50	0.00156	444.22
60	323.15	1.74	0.0184	37.65	1.48	0.00859	80.67
65	333.15	1.71	0.0164	42.27	1.46	0.00919	75.41
70	343.15	1.67	0.0203	34.14	1.45	0.00395	74.12

**Table 4:** Thermodynamic parameters of irreversible thermal denaturation of *Myceliophthora heterothallica* endoxylanase expressed in *Komagataella phaffii* with and without 20% (*v*/*v*) glycerol at 55–70 °C.

Temperature		Control			20% Glycerol		
°C	K	∆*H*_D_ (kJ·mol^−1^)	∆*G*_D_ (kJ·mol^−1^)	∆*S*_D_ (J K^−1^·mol^−1^)	∆*H*_D_ (kJ·mol^−1^)	∆*G*_D_ (kJ·mol^−1^)	∆*S*_D_ (J K^−1^·mol^−1^)
55	328.15	57.13	93.78	−111.72	116.75	98.21	56.50
60	333.15	57.09	92.92	−107.59	116.70	95.03	65.09
65	338.15	57.05	94.68	−111.34	116.66	96.31	60.22
70	343.15	57.01	95.52	−112.26	116.62	97.73	55.09

## Data Availability

The original contributions presented in this study are included in the article/Supplementary Material. Further inquiries can be directed to the corresponding author(s).

## Supplementary Materials

The following supporting information can be downloaded at: https://www.mdpi.com/article/10.3390/biotech14030062/s1, Figure S1: First-order Arrhenius plots showing the effect of temperature on the activity of Myceliophthora heterothallica endoxylanase expressed in Komagataella phaffii, during beechwood xylan hydrolysis assays. Vertical bars represent standard deviations (n = 3). (A) Free enzyme. (B) With 20% glycerol. Figure S2: First-order plots showing the effect of thermal denaturation on the activity of Myceliophthora heterothallica endoxylanase expressed in Komagataella phaffii. Enzyme samples were incubated at 40 °C (■), 50 °C (●), 60 °C (▲), and 70 °C (▼), and residual activity was determined by enzymatic assay. (A) Free enzyme. (B) With 20% glycerol. Figure S3: First-order Arrhenius plots for determining the activation energy of denaturation (Ea(D)) of Myceliophthora heterothallica endoxylanase expressed in Komagataella phaffii. (●) Free enzyme; (■) With 20% glycerol. The first-order thermal denaturation rate constants (kd) for both assay conditions were obtained from the slopes in Figures S2A and S2B.

## Abbreviations

The following abbreviations are used in this manuscript:

rMhXyn	Recombinant Myceliophthora heterothallica endoxylanase
MD	Molecular dynamics
XOS	Xylooligosaccharides
*K′*	Semisaturation constant
*V*_max_	Maximum reaction rate or velocity
*k_cat_*	Turnover number
E_a_	Activation energy
Q_10_	Temperature coefficient
T_1/2_	Half-life time
E_a(D)_	Activation energy for thermal denaturation
Δ*H*_(D)_	Enthalpy of denaturation
Δ*G*_(D)_	Gibbs free energy of denaturation
Δ*S*_(D)_	Entropy of denaturation
ES	Enzyme–substrate complex

## References

[1] Bhardwaj N., Kumar B., Verma P. (2019). A detailed overview of xylanases: An emerging biomolecule for current and future prospective. Bioresour. Bioprocess..

[2] Bastawde K.B. (1992). Xylan structure, microbial xylanases, and their mode of action. World J. Microbiol. Biotechnol..

[3] Dimarogona M., Topakas E., Christakopoulos P., Chrysina E.D. (2012). The structure of a GH10 xylanase from*Fusarium oxysporum* reveals the presence of an extended loop on top of the catalytic cleft. Acta Crystallogr. Sect. D Struct. Biol..

[4] Zhao S., Zhang G.-L., Chen C., Yang Q., Luo X.-M., Wang Z.-B., Wu A.-M., Feng J.-X. (2021). A combination of mild chemical pre-treatment and enzymatic hydrolysis efficiently produces xylooligosaccharides from sugarcane bagasse. J. Clean. Prod..

[5] Palaniappan A., Antony U., Emmambux M.N. (2021). Current status of xylooligosaccharides: Production, characterization, health benefits and food application. Trends Food Sci. Technol..

[6] Ali K., Niaz N., Waseem M., Ashraf W., Hussain M., Khalid M.U., Bin Tahir A., Raza A., Khan I.M. (2025). Xylooligosaccharides: A comprehensive review of production, purification, characterization, and quantification. Food Res. Int..

[7] Kaur G., Kaur P., Kaur J., Singla D., Taggar M.S. (2024). Xylanase, xylooligosaccharide and xylitol production from lignocellulosic biomass: Exploring biovalorization of xylan from a sustainable biorefinery perspective. Ind. Crops Prod..

[8] Gangwar A.K., Prakash N.T., Prakash R. (2014). Applicability of microbial xylanases in paper pulp bleaching: A review. BioResources.

[9] Campestrini E., Silva V.T.M.d., Appelt M.D. (2005). Utilização de enzimas na alimentação animal. Rev. Eletrônica Nutr..

[10] Yang X., Chen H., Gao H., Li Z. (2001). Bioconversion of corn straw by coupling ensiling and solid-state fermentation. Bioresour. Technol..

[11] Moretti M., Bocchini-Martins D.A., Silva R.D., Rodrigues A., Sette L.D., Gomes E. (2012). Selection of thermophilic and thermotolerant fungi for the production of cellulases and xylanases under solid-state fermentation. Braz. J. Microbiol..

[12] You S., Zha Z., Li J., Zhang W., Bai Z., Hu Y., Wang X., Chen Y., Chen Z., Wang J. (2021). Improvement of XYL10C_∆N catalytic performance through loop engineering for lignocellulosic biomass utilization in feed and fuel industries. Biotechnol. Biofuels.

[13] Stamogiannou I., Van Camp J., Smagghe G., Van de Walle D., Dewettinck K., Raes K. (2021). Impact of phenolic compound as activators or inhibitors on the enzymatic hydrolysis of cellulose. Int. J. Biol. Macromol..

[14] Hirai M., Ajito S., Sugiyama M., Iwase H., Takata S.-i., Shimizu N., Igarashi N., Martel A., Porcar L. (2018). Direct Evidence for the Effect of Glycerol on Protein Hydration and Thermal Structural Transition. Biophys. J..

[15] Ait Braham S., Siar E.H., Arana-Peña S., Bavandi H., Carballares D., Morellon-Sterling R., de Andrades D., Kornecki J.F., Fernandez-Lafuente R. (2021). Positive effect of glycerol on the stability of immobilized enzymes: Is it a universal fact?. Process Biochem..

[16] de Amo G.S., Bezerra-Bussoli C., da Silva R.R., Kishi L.T., Ferreira H., Gomes E., Bonilla-Rodriguez G.O. (2024). Heterologous expression of GH11 xylanase from *Myceliophthora heterothallica* F.2.1.4 in Pichia pastoris. Biocatal. Agric. Biotechnol..

[17] de Amo G.S., Bezerra-Bussoli C., da Silva R.R., Kishi L.T., Ferreira H., Mariutti R.B., Arni R.K., Gomes E., Bonilla-Rodriguez G.O. (2019). Heterologous expression, purification and biochemical characterization of a new xylanase from *Myceliophthora heterothallica* F.2.1.4. Int. J. Biol. Macromol..

[18] Massarente V.S., de Araujo Zanoni J., Gomes E., Bonilla-Rodriguez G.O. (2020). Biochemical characterization of endoglucanases produced by Myceliophthora thermophila M. 7.7 in solid-state culture. Biocatal. Agric. Biotechnol..

[19] Vardakou M., Dumon C., Murray J.W., Christakopoulos P., Weiner D.P., Juge N., Lewis R.J., Gilbert H.J., Flint J.E. (2008). Understanding the Structural Basis for Substrate and Inhibitor Recognition in Eukaryotic GH11 Xylanases. J. Mol. Biol..

[20] Wang J., Cieplak P., Kollman P.A. (2000). How well does a restrained electrostatic potential (RESP) model perform in calculating conformational energies of organic and biological molecules?. J. Comput. Chem..

[21] Peters G.H., Frimurer T.M., Andersen J.N., Olsen O.H. (1999). Molecular Dynamics Simulations of Protein-Tyrosine Phosphatase 1B. I. Ligand-Induced Changes in the Protein Motions. Biophys. J..

[22] Humphrey W., Dalke A., Schulten K. (1996). VMD: Visual molecular dynamics. J. Mol. Graph..

[23] Van Gunsteren W.F., Berendsen H.J.C. (1987). Gromos Manual.

[24] Ryckaert J.-P., Ciccotti G., Berendsen H.J.C. (1977). Numerical integration of the cartesian equations of motion of a system with constraints: Molecular dynamics of n-alkanes. J. Comput. Phys..

[25] Allen M.P., Tildesley D.J. (1989). Computer Simulation of Liquids.

[26] Miller G.L. (1959). Use of Dinitrosalicylic Acid Reagent for Determination of Reducing Sugar. Anal. Chem..

[27] Marangoni A.G. (2003). Enzyme Kinetics: A Modern Approach.

[28] Saqib A.A.N., Farooq A., Iqbal M., Hassan J.U., Hayat U., Baig S. (2012). A thermostable crude endoglucanase produced by *Aspergillus fumigatus* in a novel solid state fermentation process using isolated free water. Enzym. Res..

[29] Saqib A.A., Hassan M., Khan N.F., Baig S. (2010). Thermostability of crude endoglucanase from Aspergillus fumigatus grown under solid state fermentation (SSF) and submerged fermentation (SmF). Process. Biochem..

[30] Bonfá E.C., de Souza Moretti M.M., Gomes E., Bonilla-Rodriguez G.O. (2018). Biochemical characterization of an isolated 50 kDa beta-glucosidase from the thermophilic fungus Myceliophthora thermophila M. 7.7. Biocatal. Agric. Biotechnol..

[31] Trindade L.V., Desagiacomo C., Polizeli M.d.L.T.d.M., Damasio A.R.d.L., Lima A.M.F., Gomes E., Bonilla-Rodriguez G.O. (2016). Biochemical characterization, thermal stability, and partial sequence of a novel exo-polygalacturonase from the thermophilic fungus Rhizomucor pusillus A13. 36 obtained by submerged cultivation. BioMed Res. Int..

[32] Siddiqui K.S., Saqib A.A.N., Rashid M.H., Rajoka M.I. (1997). Thermostabilization of carboxymethylcellulase from Aspergillus niger by carboxyl group modification. Biotechnol. Lett..

[33] Vucinic J., Novikov G., Montanier C.Y., Dumon C., Schiex T., Barbe S. (2021). A Comparative Study to Decipher the Structural and Dynamics Determinants Underlying the Activity and Thermal Stability of GH-11 Xylanases. Int. J. Mol. Sci..

[34] Choi J.M., Seo M.-H., Kyeong H.-H., Kim E., Kim H.-S. (2013). Molecular basis for the role of glucokinase regulatory protein as the allosteric switch for glucokinase. Proc. Natl. Acad. Sci. USA.

[35] Oliveira I.C.M., Garay A.V., Souza A.A., Valadares N.F., Barbosa J.A.R.G., Faria F.P., Freitas S.M. (2022). Structural and biochemical analysis reveals how ferulic acid improves catalytic efficiency of *Humicola* grisea xylanase. Sci. Rep..

[36] Moreira L.R.d.S., Campos M.d.C., de Siqueira P.H.V.M., Silva L.P., Ricart C.A.O., Martins P.A., Queiroz R.M.L., Filho E.X.F. (2013). Two β-xylanases from Aspergillus terreus: Characterization and influence of phenolic compounds on xylanase activity. Fungal Genet. Biol..

[37] Mwangi I.W., Ngila J.C., Ndung’U P., Msagati T.A. (2014). Removal of phenolics from aqueous media using quaternised maize tassels. J. Environ. Manag..

[38] Costa T.d.S., Rogez H., Pena R.d.S. (2015). Adsorption capacity of phenolic compounds onto cellulose and xylan. Food Sci. Technol..

[39] Borjian N., Farhadian S., Shareghi B., Asgharzadeh S., Momeni L., Ghobadi S. (2025). Binding affinity and mechanism of dicofol-lysozyme interaction: Insights from multi-spectroscopy and molecular dynamic simulations. Int. J. Biol. Macromol..

[40] Driss D., Bhiri F., Ghorbel R., Chaabouni S.E. (2012). Cloning and constitutive expression of His-tagged xylanase GH 11 from Penicillium occitanis Pol6 in Pichia pastoris X33: Purification and characterization. Protein Expr. Purif..

[41] Wang L., Cao K., Pedroso M.M., Wu B., Gao Z., He B., Schenk G. (2021). Sequence- and structure-guided improvement of the catalytic performance of a GH11 family xylanase from Bacillus subtilis. J. Biol. Chem..

[42] Deponte M., Sturm N., Mittler S., Harner M., Mack H., Becker K. (2007). Allosteric Coupling of Two Different Functional Active Sites in Monomeric *Plasmodium falciparum* Glyoxalase I. J. Biol. Chem..

[43] Vagenende V., Yap M.G., Trout B.L. (2009). Mechanisms of Protein Stabilization and Prevention of Protein Aggregation by Glycerol. Biochemistry.

[44] Gajardo-Parra N.F., Akrofi-Mantey H.O., Ascani M., Cea-Klapp E., Garrido J.M., Sadowski G., Held C. (2022). Osmolyte effect on enzymatic stability and reaction equilibrium of formate dehydrogenase. Phys. Chem. Chem. Phys..

[45] Tamoliūnas K., Galamba N. (2020). Protein Denaturation, Zero Entropy Temperature, and the Structure of Water around Hydrophobic and Amphiphilic Solutes. J. Phys. Chem. B.

[46] Saridakis E., Donta K. (2024). Protein Thermodynamic Properties, Crystallisation, and the Hofmeister Series. Chempluschem.

[47] Shostka V., Shostka N., Halilov S., Vershitsky V. (2020). Formation of clathrate structures in surface layers of heavily diluted aqueous solutions. Journal of Physics: Conference Series.

[48] Sheng Y., Lam S.S., Wu Y., Ge S., Wu J., Cai L., Huang Z., Van Le Q., Sonne C., Xia C. (2021). Enzymatic conversion of pretreated lignocellulosic biomass: A review on influence of structural changes of lignin. Bioresour. Technol..

[49] Manna B., Ghosh A. (2021). Understanding the conformational change and inhibition of hyperthermophilic GH10 xylanase in ionic liquid. J. Mol. Liq..

