# Supplementary material for: Effects of Glycerol and Phenolics on Myceliophthora heterothallica Endoxylanase Expressed in K. phaffii
Source: BioTech (Basel). 2025 Aug 18;14(3):62. doi: 10.3390/biotech14030062 (PMC12372066; doi:10.3390/biotech14030062)
Supplement: Supplementary file 1 [file biotech-14-00062-s001.zip › biotech-3731676-supplementary.pdf]

BioTech

MDPI

Supplementary material

Effects of Glycerol and Phenolics on *Myceliophthora heterothallica* Endoxylanase Expressed in *K. phaffii*

Jéssica de Araujo Zanoni[1], Izabela Karolina Costa Zilli[1], Guilherme de Paula Pretto[1], Flavio Augusto Vicente Seixas[2], Marcela Marques de Freitas Lima[3], Eliana Gertrudes de Macedo Lemos[4], Eleni Gomes[3], Gabriel Zazeri[5]* and Gustavo Orlando Bonilla-Rodriguez[3]*

[1] Graduate Program in Microbiology, Institute of Biosciences, Humanities and Exact Sciences, São Paulo State University, IBILCE-UNESP, São José do Rio Preto, SP, Brazil 15054-000; j.zanoni@unesp.br (J.d.A.Z.); izabela.kc.zilli@unesp.br (I.K.C.Z.); g.pretto@unesp.br (G.d.P.P.)
[2] Department of Technology, State University of Maringá (UEM), Umuarama, PR, Brazil 87020-900; favseixas@uem.br
[3] Department of Chemistry and Environmental Sciences, Institute of Biosciences, Humanities and Exact Sciences, São Paulo State University (IBILCE-UNESP), São José do Rio Preto, SP, Brazil 15054-000; marcela-marques.lima@unesp.br (M.M.d.F.L.); eleni.gomes@unesp.br (E.G.)
[4] Department of Technology, School of Agricultural and Veterinarian Sciences, São Paulo State University (FCAV-UNESP). Jaboticabal, SP, Brazil 14884-900; eliana.lemos@unesp.br
[5] Department of Physics, Federal University of Roraima (UFRR), Boa Vista, RR, 69310-000, Brazil ;
* Correspondence: gabriel.zazeri@ufrr.br (G.Z.); gustavo.b.rodriguez@unesp.br (G.O.B.-R.)

The optimal temperature of *Myceliophthora heterothallica* endoxylanase expressed in *Komagataella phaffii*, under optimal pH conditions, was estimated (Equation S1) from the x-axis intercept at the intersection between the ascending and descending regions of the Arrhenius plot (Figure S1).

Equation S1: $T(ºC) = (\frac{1000}{x}) - 273{,}15$

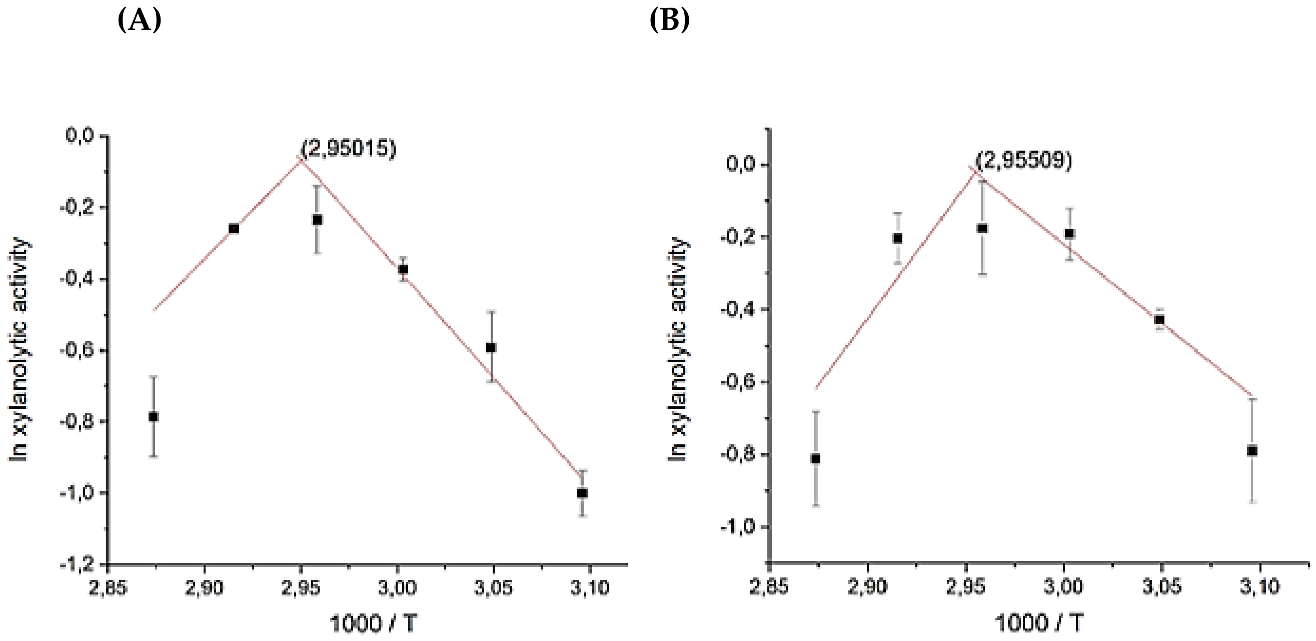

 https://doi.org/10.3390/xxxxx

Figure S1. First-order Arrhenius plots showing the effect of temperature on the activity of *Myceliophthora heterothallica* endoxylanase expressed in *Komagataella phaffii,* during beechwood xylan hydrolysis assays. Vertical bars represent standard deviations (n = 3). (A) Free enzyme. (B) With 20% glycerol.

The activation energy (Ea) was calculated using Equation S2, where *R* is the universal gas constant.

Equation S2: $Ea = -(slope.R)$

The activation energy value was then used to determine the temperature coefficient (Q_{10}) for each temperature according to Equation S3.

Equation S3: $ln\, Q_{10} = \frac{(Ea\,.10)}{(R.T^2)}$

For determination of thermodynamic parameters, the enzyme was incubated in the absence of substrate at various temperatures for different time intervals. Samples were then kept at 4 °C to allow possible refolding of the native structure before measuring residual activity, which was used to calculate the rate of activity loss.

Half-life ($T_1/_2$) values were calculated using Equation S4, where k_d values were obtained from the negative slopes of the linear regressions shown in Figures S2A and S2B.

Equation S4: $T_{1/2} = ln\,\frac{2}{k_d} = \frac{0{,}693}{k_d}$,

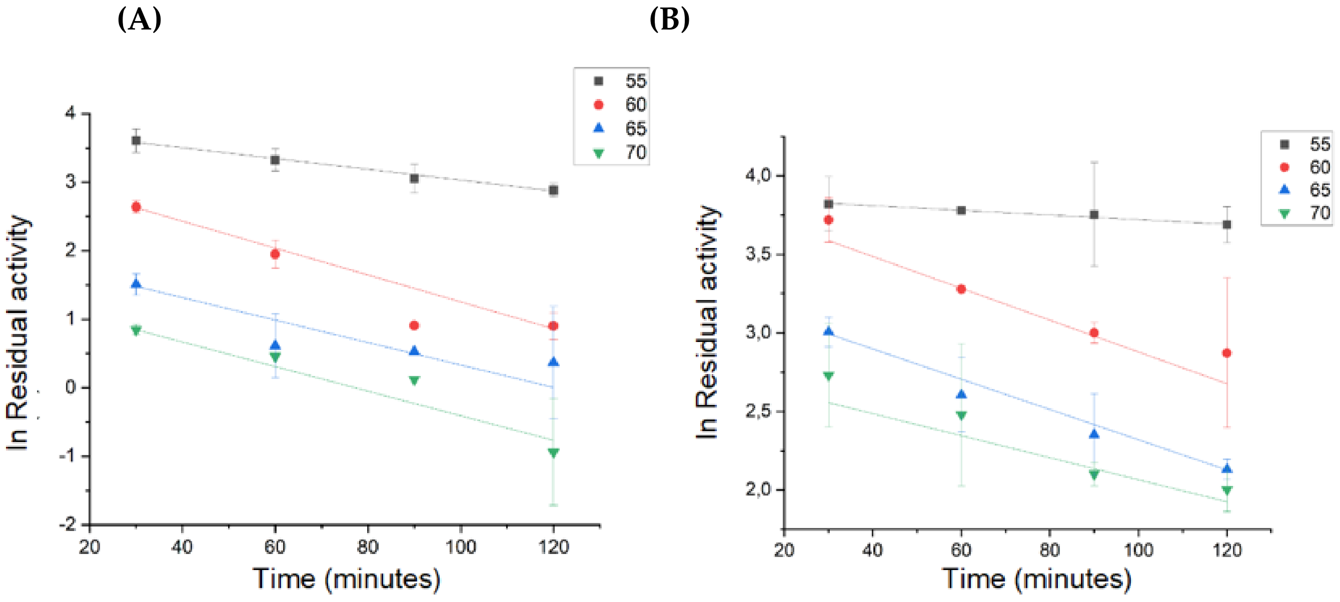

Figure S2. First-order plots showing the effect of thermal denaturation on the activity of *Myceliophthora heterothallica* endoxylanase expressed in *Komagataella phaffii.* Enzyme samples were incubated at 40 °C (■), 50 °C (●), 60 °C (▲), and 70 °C (▼), and residual activity was determined by enzymatic assay. (A) Free enzyme. (B) With 20% glycerol.

The k_d values (min^{-1}) obtained for each temperature were used to construct the plot shown in Figure S3, which provided the data required to calculate the activation energy of thermal denaturation (Equation S5) and, subsequently, the other thermodynamic parameters.

Figure S3. First-order Arrhenius plots for determining the activation energy of denaturation (Ea(D)) of *Myceliophthora heterothallica* endoxylanase expressed in *Komagataella phaffii*. (●) Free enzyme; (■) With 20% glycerol. The first-order thermal denaturation rate constants (k_d) for both assay conditions were obtained from the slopes in Figures S2A and S2B.

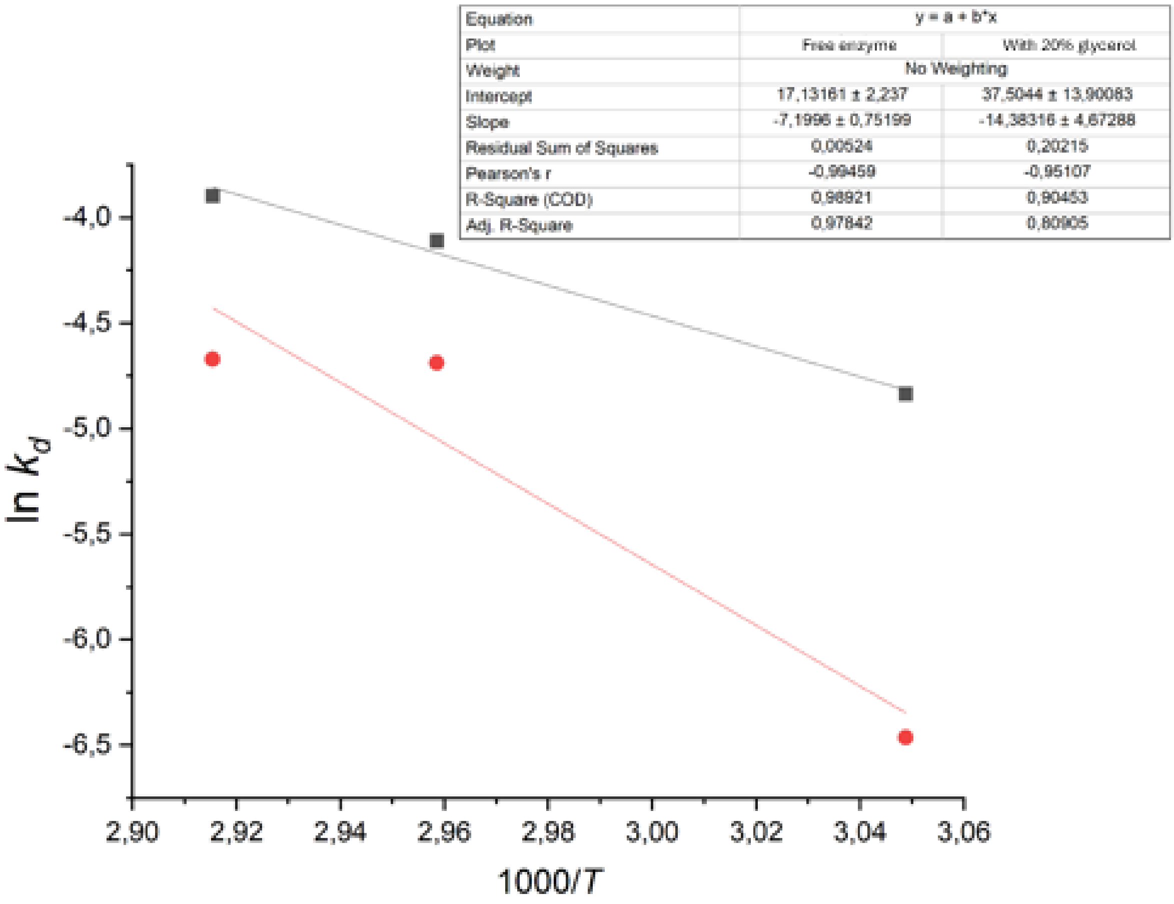

Equation	y = a + b*x	
Plot	Free enzyme	With 20% glycerol
Weight	No Weighting	
Intercept	17,13161 ± 2,237	37,5044 ± 13,90083
Slope	-7,1996 ± 0,75199	-14,38316 ± 4,67288
Residual Sum of Squares	0,00524	0,20215
Pearson's r	-0,99459	-0,95107
R-Square (COD)	0,98921	0,90453
Adj. R-Square	0,97842	0,80905

The thermodynamic parameters: Denaturation activation energy ($E_{a(D)}$); Enthalpy (ΔH_D (kJ mol^{-1})); Gibbs free energy (ΔG_D (kJ mol^{-1})); and Entropy (ΔS_D (J K^{-1} mol^{-1})), were calculated following the equations listed below:

Equation S5: $E_{a(d)} = -(slope.R)$

Equation S6: $\Delta H_D = E_{a(d)} - R.T$

Equation S7: $\Delta G_D = -R.T \times ln\left(\frac{k_d \times h}{k_b \times T}\right)$

k_b: Boltzmann constant = 1.38 x 10^{-23} J K^{-1}

h: Planck constant = 6,63 x 10^{-34} J s

Equation S8: $\Delta S_D = \frac{(\Delta H_D - \Delta G_D)}{T}$

Disclaimer/Publisher's Note: The statements, opinions and data contained in all publications are solely those of the individual author(s) and contributor(s) and not of MDPI and/or the editor(s). MDPI and/or the editor(s) disclaim responsibility for any injury to people or property resulting from any ideas, methods, instructions or products referred to in the content.
